# Near-field spectroscopic investigation of dual-band heavy fermion metamaterials

**DOI:** 10.1038/s41467-017-02378-3

**Published:** 2017-12-22

**Authors:** Stephanie N. Gilbert Corder, Xinzhong Chen, Shaoqing Zhang, Fengrui Hu, Jiawei Zhang, Yilong Luan, Jack A. Logan, Thomas Ciavatti, Hans A. Bechtel, Michael C. Martin, Meigan Aronson, Hiroyuki S. Suzuki, Shin-ichi Kimura, Takuya Iizuka, Zhe Fei, Keiichiro Imura, Noriaki K. Sato, Tiger H. Tao, Mengkun Liu

**Affiliations:** 1 Department of Physics and Astronomy, Stony Brook University, Stony Brook, NY 11794 USA; 2 Department of Mechanical Engineering, University of Texas-Austin, Austin, TX 78712 USA; 3 Department of Physics and Astronomy, Iowa State University, Ames, IA 50011 USA; 4 Advanced Light Source Division, Lawrence Berkeley National Laboratory, Berkeley, CA 94720 USA; 5 Department of Physics and Astronomy, Texas A&M University, College Station, TX 77843 USA; 6 National Institute for Materials Science, Tsukuba, 305-0047 Japan; 7 UVSOR Facility, Institute for Molecular Science, Okazaki, 444-8585 Japan; 8 Department of Physics, Nagoya University, Nagoya, Japan; 9 Present Address: State Key Laboratory of Transducer Technology, Shanghai Institute of Microsystem and Information Technology, Chinese Academy of Sciences, Shanghai, 200050 China; 10 Present Address: Institute for Solid State Physics, University of Tokyo, Kashiwa, 277-8581 Japan; 11 Present Address: Graduate School of Frontier Biosciences and Department of Physics, Osaka University, Suita, Osaka, 565-0871 Japan; 12 Toyota Technological Institute, Nagoya, 468-8511 Japan

## Abstract

Broadband tunability is a central theme in contemporary nanophotonics and metamaterials research. Combining metamaterials with phase change media offers a promising approach to achieve such tunability, which requires a comprehensive investigation of the electromagnetic responses of novel materials at subwavelength scales. In this work, we demonstrate an innovative way to tailor band-selective electromagnetic responses at the surface of a heavy fermion compound, samarium sulfide (SmS). By utilizing the intrinsic, pressure sensitive, and multi-band electron responses of SmS, we create a proof-of-principle heavy fermion metamaterial, which is fabricated and characterized using scanning near-field microscopes with <50 nm spatial resolution. The optical responses at the infrared and visible frequency ranges can be selectively and separately tuned via modifying the occupation of the 4f and 5d band electrons. The unique pressure, doping, and temperature tunability demonstrated represents a paradigm shift for nanoscale metamaterial and metasurface design.

## Abstract (web summary)

Understanding the electromagnetic responses at subwavelength scales is important for achieving tunability. Using a combination of the near-field and far-field spectroscopy, the authors demonstrate a heavy fermion metamaterial with tunable dual-band optical responses by selectively and separately modifying the 4f and 5d band electrons.

## Introduction

Typically composed of composite systems with artificial subwavelength structures or inclusions, metamaterials (MM) were first introduced to supplement or replace naturally occurring solids where the electromagnetic response is limited by the inherent properties resulting from the electronic band structure^1–3^. Recent developments, however, have embraced a reverse trend in which the intrinsic optical properties of natural materials are employed to aid in the design of new generations of MM devices^4^. This is especially relevant in the construction of switchable or tunable MM with dynamic control. For example, MM hybridized with phase-change materials such as transition metal oxides (vanadium oxides)^5,6^, tunable 2D materials (graphene)^7^, and chalcogenide glasses (Ge_2_Sb_2_Te_5_)^8^ have been explored extensively for this cause.

One major subset of phase-change materials is the strongly correlated electron systems (SCES), in which the competing electron–electron and electron–lattice interactions result in a high susceptibility to small external stimuli. A massive redistribution of the spectral weight over a broad frequency range can occur in SCES under strain, doping, or thermal excitation. So far, tunable MM design has mainly focused on altering/tuning the response of noble-metal resonators in composite devices by relying heavily on the single band electron properties of the SCES as functional substrates^5,6,8–10^.

In this work, we demonstrate a unique route to creating dual-band plasmonic MM comprising a single strongly correlated heavy fermion system. We control and monitor the plasmonic response of not only d-band electrons, as in noble metals or transition metal oxides, but also of f-band electrons. This is achieved by utilizing the local phase transition in samarium sulfide (SmS), a canonical correlated heavy fermion system with 5d and 4f electrons. The application of modest pressure (6.5 kbar^11,12^) turns the optically black semiconductor into a golden semi-metal and is accompanied by a broad spectral weight redistribution^13,14^. By taking advantage of this pressure-dependent insulator to metal phase transition (IMT), we are able to write a conducting pattern on the material surface using an atomic force microscope (AFM) in ambient conditions without the need of a lithographic mask, metal deposition, extended sample preparation, or high vacuum requirements. Through controlled local distribution of pressure at the nanoscale and MM design, we are able to alter carrier occupations of the two energy bands and engineer the effective carrier density at the sample surface, giving rise to plasmonic resonances in the IR and visible regions. A combination of near-field and far-field optical techniques enables us to characterize the nanoscale phase separation and probe the electromagnetic response of the resulting devices from submicroscopic to macroscopic scales.

## Results

### Origin and spatial engineering of the dual-band resonances

**Fig. 1:**
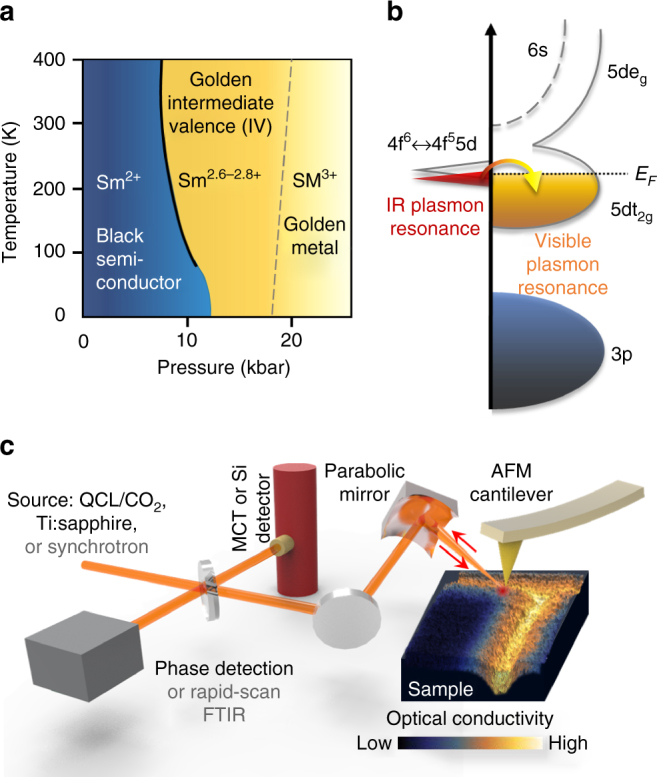
Material properties of SmS and a schematic of the nano-spectroscopy and nano-imaging setup. **a** Phase diagram of samarium sulfide (SmS) as a function of pressure and temperature. The material undergoes a black to golden optical phase transition as the occupation of the valence states of the material change with applied pressure. **b** Diagram of golden IV phase of SmS after Batlogg et al.^11^. The IR plasmon resonance is attributed to carriers in the 4f states, while the visible resonance is due to 5d carriers^16^. As pressure increases, the 4f electrons move into the 5d t_2g_ state as indicated by the arrow^17^. **c** AFM nano-lithography is performed on the SmS sample surface, producing a region of controlled strain defined by the AFM tip radius. The resulting pattern (shown in false color) is subsequently probed with scattering-scanning near-field optical microscopy (s-SNOM) to measure the optical changes. A laser-based or synchrotron light source is used to probe the near-field optical response at visible or IR frequencies

The phase diagram of SmS as a function of pressure and temperature is shown schematically in Fig. 1a (after^14,15^). At ambient pressure, the semiconducting black phase of SmS has a direct band gap of ~0.4 eV and an indirect gap of ~0.09 eV^13,15^. The Sm 4f^6^ states are highly localized such that the overlap between these electron wavefunctions vanishes and no plasma resonance exists in spite of the high 4f electron concentration^16^. With increasing pressure (crystal field splitting), SmS undergoes an isostructural phase transition to a golden intermediate valence (IV) state: from Sm^2+^ to a fractional valence of Sm^2.6−2.8+^. This intermediate valence is a result of the 5d t_2g_ conduction band shifting lower in energy and hybridizing with the 4f^6^ band on the neighboring atomic site. As the valence changes, carriers move from the highly localized 4f^6^ state into the 5d t_2g_ conduction band^17^. These former f electrons (now in the d band) have residual interactions with the holes left behind, producing excitonic correlations^18^. In the golden IV phase, two distinct plasmon resonances exist, corresponding to light d (visible plasmon mode) electrons and heavy f (IR plasmon mode) electrons, shown schematically in Fig. 1b ^16^. Above 20 kbar, the system becomes fully metallic (Sm^3+^), the 4f resonance disappears as the gap collapses, and the 5d t_2g_ band is filled with 4f electrons^17^. For more details on the material properties, please see Supplementary Note 1.

In order to transform a bulk SmS single crystal into a reflective MM, we use contact mode AFM lithography to fabricate a series of surface structures. A conventional AFM tip writes a pre-defined pattern with controllable local pressure of ~5–15 kbar (calculated from force–distance curves) with 20 nm precision and 50 nm linewidth, as schematically illustrated in Fig. 1c and discussed in Supplementary Notes 2 and 3. This induces a local IMT in SmS, resulting in a color change from black to golden together with a large spectral weight shift in the visible and IR^13,14^. The metallic volume fraction on the material surface can thus be selectively modified from 0 to 100%, facilitating nanoscale pattern formation. In contrast to previous demonstrations of “sketching” oxide heterostructures with voltage pulses^19^, the method discussed here does not require the fabrication of electrodes or the application of an electrical voltage. Scattering-type scanning near-field optical microscopy (s-SNOM) techniques are sequentially employed to investigate the strain-induced IMT at the nanoscale. The s-SNOM method enables nano-imaging^20^ and nano-spectroscopy^21,22^ which can locally probe (with resolution on the order of the AFM tip diameter) the resultant optical response of the tip-patterned region, thus verifying the presence of the metallic volume fraction at the sample surface after tip lithography (Fig. 1c). See Supplementary Note 4 for further details on the s-SNOM technique.

### Near-field characterization of the dual-band resonances

**Fig. 2:**
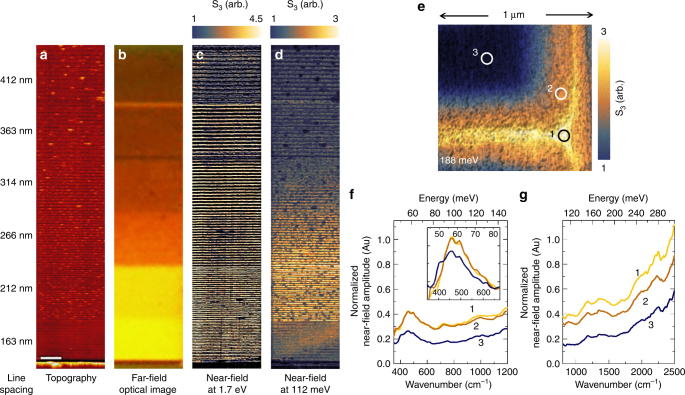
IR and visible near-field characterization of SmS metamaterials created via AFM lithography. **a** The topography of a color gradient MM with decreasing spacing between the patterned lines (listed on the left) is shown for a depth of ~12 nm. The scale bar is 500 nm. A far-field optical image of the same gradient MM is shown in **b**; the color change from red to golden is achieved by altering the lithographic line spacing. Room-temperature near-field images are collected at 1.7 eV (**c**), and 112 meV (**d**). The near-field 3rd harmonic amplitude is shown in false-color, where blue indicates regions of semiconducting conductivity and gold indicates regions of metallic conductivity. **e** The near-field amplitude at 188 meV (1515 cm^−1^) and 295 K is shown in false color for a 1 μm^2^ region of a grating pattern. The near-field amplitude change of the patterned area (in gold) is normalized to the semiconducting response (in blue). Three locations are marked in the image, indicating golden IV (1), transition (2), and semiconducting (3) regions of the MM. Broadband near-field spectroscopy (normalized to a gold thin film) at 295 K is shown in **f**, **g**, for the locations specified in the 1 μm^2^ image, indicating the presence of the localized golden IV phase on the surface of the SmS. The exciton peak at 58 meV (468 cm^−1^) is observable in all three regions. The inset in **f**, shows the exciton peaks with the background removed to highlight the slight frequency shift with applied pressure

Using AFM lithography, we pattern a gradient MM with decreasing line spacing from 412 to 163 nm as seen in the topography image of Fig. 2a. A visible red-golden color gradient can be immediately observed following the lithography process under a far-field white light camera (Fig. 2b). To understand the origin of this macroscopic color change, subsequent near-field imaging at 1.7 eV is performed to probe the contribution of the 5d t_2g_ band electrons. As shown in Fig. 2c, the blue and golden colors indicate semiconducting and conductive responses, respectively. The unpatterned regions above and below the MM show little signal (and indeed between the lithographic lines as well), confirming the lack of free carriers in the 5d conduction band prior to patterning. With the exception of the largest spacing (442 nm), the near-field signal is consistent for all spacings at 1.7 eV, indicating the change in the visible color in the far-field image (therefore areal averaged free carrier density) is solely determined by the line spacing rather than an actual gradient in the occupation of the d-electron band. In contrast, IR near-field imaging reveals a local electronic response different from that at 1.7 eV: at 112 meV, for example, the highest near-field signal is not at the lower end of the grating MM but one color step above, as shown in Fig. 2d. At 112 meV, we are monitoring the delocalization of the 4f electrons while in the visible range only the 5d electrons are probed. In order to better understand the IR near-field response, we also perform IR broadband nano-spectroscopy as detailed below.

Figure 2e shows the near-field amplitude (S_3_) image of a 1 μm^2^ region of a grating MM at 188 meV (1515 cm^−1^). The surface nano-structure is probed with ultra-broadband nano-spectroscopy in three 20 nm locations designated in Fig. 2e: the region of maximum local strain (1), a transition region adjacent to the maximum strain (2), and an unstrained region (3). The results are shown in Fig. 2f, g, where the broadband near-field amplitude is normalized to a thin film gold reference^22,23^. The lithographically strained regions (1) and (2) show an increase in far infrared reflectivity, indicating the patterned areas have a stronger broadband metallic response when compared to the semiconducting unstrained region (3). The peak in Fig. 2f around 58 meV (468 cm^−1^) is attributed to an exciton near the indirect bandgap of SmS (around 87 meV or 700 cm^−1^)^13^ and the gentle slope at higher frequencies indicates the onset of the 4f^6^–5d t_2g_ absorption edge^11^. The inset in Fig. 2f illustrates the shift of the exciton peak to slightly higher frequencies^13^ and the baseline reflectivity increases significantly with the lithographic strain: the application of pressure causes the 5d t_2g_ band to shift lower in energy relative to the 4f band, and f electrons are transferred into the hybridized state, increasing the baseline reflectivity without changing the spectral features^13,17,24^. The small peaks present between 700 and 2500 cm^−1^ are likely intra-4f^5^ transitions, which become increasingly infrared active with applied pressure^16^. The presence of the exciton peak and indirect band gap between the 4f^6^ and 5d t_2g_ bands indicates that the golden phase produced by the patterning is still in the IV regime, and therefore we have access to plasmon resonances in both the 4f and 5d energy bands. This is not immediately clear from the fabrication process as the fully metallic and IV states are identical at visible frequencies—the plasmon resonance of the 5d conduction band gives rise to the golden color and is present in both IV and fully metallic SmS.

In Fig. 2e–g, the existence of a transition region demonstrates that the effects of the strain on the lattice extend beyond the lithography. Therefore, we attribute the increase in signal strength as line spacing decreases from 412–212 nm in Fig. 2d to an accumulation of local strain, which produces increasing overlap of the 4f electronic states and results in a stronger plasmonic resonance up to a threshold. As the population of carriers in the 4f band is reduced with increasing pressure, the strength of the 4f resonance ultimately decreases as can be seen in Fig. 2d for the 163 nm spacing. Since the depopulation of the 4f band occurs as a function of local strain and not necessarily at the same location as the smallest line spacing, the optical responses in the visible and IR range can be modulated independently—this will be further explored with a doped SmS MM. Therefore, we conclude that the far-field color gradient primarily arises from the differences in line spacing, resulting in the observed golden, orange, and red optical colors. The 4f electron plasmonic resonance does not play a role in determining the apparent color, but displays a strong dependence on the local strain.

### Far-field characterization of the dual-band resonances

**Fig. 3:**
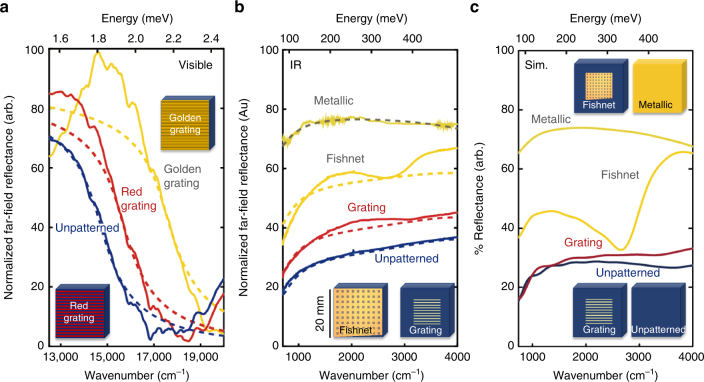
Tuning the far-field response of SmS metamaterials in the visible and IR range. **a** Reflectance spectra (solid lines) of golden visible MM grating and red visible MM grating, compared to an unpatterned semiconducting reference at 295 K. The data are fit (dashed lines) using the Drude dielectric model, which shows the shift of the plasma edge. **b** IR spectra (solid lines) of a fishnet and grating MM as well as reference unpatterned semiconducting and metallic (golden) surfaces at 295 K. The data are fit using effective medium theory (dashed lines), indicating the overall reflectance change is a function of metallic fill fraction. Schematics of the 20 × 20 μm regions of the fishnet (lower left) and grating (lower right) are shown in the inset. **c** CST simulations of the experimental data in **b**, reproducing the peaks observed in the MM structures. Schematics of the four simulated regions are shown as insets

Broadband control of the visible plasmon resonance is demonstrated through effective carrier density-tuning of the far-field MM response in Fig. 3a. By adjusting the periodicity of the lithographic lines, we tune the visible reflectivity of the MM from gray (unpatterned semiconducting) to red or golden. The reflectivity of golden MM grating has a strong response in the yellow and orange portions of the spectrum (~1.98–2.2 eV), but drops quickly in the red region (~1.6 eV), giving rise to the golden color. The red MM grating is highly reflective below 1.8 eV. The reflectivity minima observed in all three spectra are due to the phonon-coupled plasmon edge, which also produces the steep reflectivity change in the visible^11,14,16^. Details of the far-field measurements are discussed in Supplementary Notes 5 and 6. The spectra are fit with the Drude model of the dielectric function (dashed curves in Fig. 3; the model parameters can be found in Supplementary Note 7) to illustrate the plasma frequency shift to higher frequencies with increased lithographic line periodicity; indicating the effective free carrier population, *N*, is increasing. The effective plasma frequency values *ω*_p_ obtained from the Drude fits are 1.81 eV (14,600 cm^−1^), 1.93 eV (15,550 cm^−1^), and 2.16 eV (17,450 cm^−1^) for the unpatterned semiconductor, red MM, and golden MM spectra, respectively. The fully metallic golden *ω*_p_ value is reported to be 2.45 eV^11^.

In addition to pressure tuning, the broadband IR reflectivity can be altered by changing the metallic fraction within the MM as well. To demonstrate, four distinct 20 × 20 μm regions are investigated in Fig. 3b (solid lines): an unpatterned semiconducting region, a polished metallic region, and two lithographically patterned structures—fishnet and grating MM. The fishnet MM consists of a 20 × 20 lithographic grid with line spacing of 1 μm and a total area of ~40 × 40 μm, while the grating MM is 10 parallel lines spaced 1 μm apart with a total area of ~12 × 12 μm. Area-averaged far-field reflectance spectroscopy over a broad spectral range is performed using a gold film as a reference. The unpatterned semiconducting and metallic responses are consistent with previous reflectance measurements^11,16^. In the semiconducting response, the reflectivity minimum at the low-energy side is the result of a plasma resonance coupled to an LO phonon mode^11^. The presence of the tip-engineered MM dramatically increases the IR reflectance; further, a broad resonance appears centered near 0.21 eV (1700 cm^−1^) in both MM while an additional resonance is evident at 0.5 eV (4000 cm^−1^) for the fishnet only. The reflectance response of the four regions is simulated using Lichtenecker’s mixing rule based on the effective medium theory (EMT) for surface inclusions (Fig. 3b: dashed lines, see Supplementary Note 8 for details on the simulation method and parameters) that takes into consideration the metallic fill fraction as well as the shape of the metallic inclusions. The EMT fit shows good agreement with all of the far-field reflectance spectra, verifying that the broadband reflectivity increase is the result of the increased metallic volume fraction within the measurement area.

The IR resonance peaks, attributed to plasmonic shape effects, are characterized by simulating the reflectance in CST Microwave Studio as discussed in Supplementary Note 9. For the grating MM, the metallic area is an accurate representation of the experimental parameters. The simulated metallic area of the fishnet is smaller than the experimental conditions in order to save computational time, and the baseline reflectivity offset of the simulated fishnet response has been shifted up to account for the decrease in total metallic surface area compared to the actual MM pattern. The models reproduce these plasmonic resonances quite well, illustrating the two absorption features arise from the geometry of the MM.

### Dopant and temperature tuning of the heavy fermion band

**Fig. 4:**
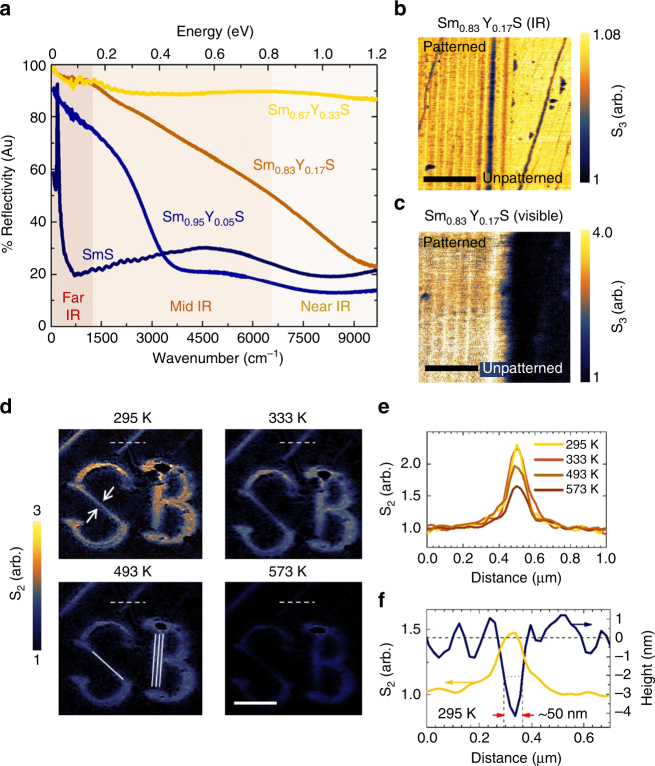
Dopant and temperature tuning of the near-field reflectivity. **a** Far-field reflectivity plot of Sm_1−*x*_Y_*x*_S for *x* = 0, 0.05, 0.17, and 0.33. **b**, **c** Near-field imaging of a grating pattern on the surface of a Sm_0.83_Y_0.17_S sample collected at 113 meV and 1.68 eV, respectively. The scale bars in both images are 1 μm. **d** Near-field imaging at 117 meV of micro-letters “SB” (Stony Brook) patterned on the surface of undoped SmS at room temperature subsequent to heat treatment at 333, 493, and 573 K. The solid white lines in the 493 K image indicate the number of lithographic lines required to produce the pattern in given areas. The scale bar is 1 μm. **e** A direct comparison of the near-field amplitude line profiles indicated by dashed white lines in **d**, is shown, highlighting the ability to tune the strength of the local IR reflectivity with temperature. **f** Height profile (blue line) and normalized near-field amplitude (gold line) of a lithographically patterned line (indicated by white arrows) at the center of the lithographic “S” in **d**. The FWHM is ~50 nm

Finally, in Fig. 4, we demonstrate the ability to tune the strain-engineered plasmonic responses with doping and temperature. Substitutional yttrium (Y) alters the optical properties of SmS, as can be seen in the far-field FTIR reflectivity spectra of Fig. 4a for Sm_1−*x*_Y_*x*_S for *x* = 0–33%. In undoped SmS, the 4f electron band is noticeable as a sharp resonance in the far IR. With increasing yttrium doping, this resonance essentially disappears as the 4f band merges with the 5d conduction band, consistent with previous reports^11,25^. Figure 4b and c show near-field images collected on a patterned yttrium-doped sample Sm_0.83_Y_0.17_S in the IR and visible frequency ranges, respectively. In the IR image, collected at 113 meV, there is essentially no conductivity change between the patterned and unpatterned regions, in marked contrast to what was observed in the undoped SmS in Fig. 2d. On the other hand, the visible conductivity change between the patterned regions measured at 1.68 eV is as strong or stronger than what was observed in the undoped SmS sample in Fig. 2c. This ability to turn off the susceptibility of the IR resonance to pressure while maintaining or strengthening the visible is a direct result of the multi-band nature of heavy fermion compounds: the yttrium substitution alters the 4f electron levels, causing them to merge with the 5d conduction band^26,27^.

The IR near-field images of the pre-patterned letters SB (initials of Stony Brook) are taken at 295 K after heat treatment at temperatures ranging from 295 to 573 K as shown in Fig. 4d. With increasing heat treatment temperature, it is clear that the IR contrast of the “SB” pattern at 117 meV is gradually disappearing. Figure 4e compares the near-field amplitude at the same location (indicated by dashed white lines) for four heat treatment temperatures, confirming the decrease in optical reflectivity. We attribute this thermal tunability to the reduced 4f–5d orbital overlap resulting from lattice expansion, which causes the system to return to the semiconducting phase^14^. Figure 4f compares the topography and near-field amplitude across the same lithographically fabricated line at room temperature (295 K), indicating the average FWHM of a typical patterned line before heat treatment. This ability to systematically reduce the reflectivity with thermal treatment opens the possibility of controlling the MM performance after the fabrication process is complete.

## Discussion

In summary, our work demonstrates that the combination of ultra-broadband near-field and far-field characterization is appealing for studying correlated nanophotonics. The fine spatial resolution provided by s-SNOM enables the measurement of excitons at room temperature in the intermediate valence golden phase as well as incremental increases in the absorption edge across the black to golden transition, indicative of strongly localized 4f electrons moving into the 5d t_2g_ band. Access to the near- and far-field responses enables the ability to separately identify and tune the f and d electron contributions to the MM response. Further, substitutional doping of yttrium in place of the samarium ions makes it is possible to effectively turn off the 4f plasmon resonance, leaving only the 5d electron resonance. Therefore, by strategically utilizing phase diagrams, highly tunable photonic devices can be created in correlated poor metals with extreme flexibility over a broad spectral range. This methodology can be generally applied to other pressure sensitive SCES such as Mott insulators (V_2_O_3_^28^, Ca_2_RuO_4_^29^), and high-*T*_c_ superconductors^30^, where noble-metal structures can be incorporated into future designs to compensate for the losses induced by the heavy electrons.

## Methods

### Sample growth, patterning, and strain

The single-crystal materials were grown by the vertical Bridgman method in a high-frequency induction furnace. Further details on the growth and materials characterization can be found in Supplementary Note 1. The AFM lithography is discussed in Supplementary Note 2. For information on the applied force during AFM patterning and residual strain estimates, please see Supplementary Note 3.

### Near-field characterization

To collect the near-field images and spectra, the following systems were used: NeaSpec NeaSNOM, NT-MDT NTEGRA-IR, and the Synchtrotron Infrared Nano-Spectroscopy (SINS) beamline 5.4 at the Advanced Light Source, Lawrence Berkeley National Laboratory. For the images, the metallic (patterned) signal was referenced to the semiconducting (unpatterned) response. The broadband spectra collected at ALS were normalized to a gold thin film. Details on the specific experimental near-field systems, along with information on the tuning of near-field properties with doping and temperature can be found in Supplementary Note 4.

### Far-field characterization

Far-field microscopy at visible frequencies was performed with an optical microscope coupled to a fiber spectrometer. An FTIR at beamline 5.4 of the Advanced Light Source, Lawrence Berkeley National Laboratory, was used to determine the broadband IR response. Details on both can be found in Supplementary Notes 5 and 6.

### Simulations

Supplementary Notes 7–9 contain details on the Drude and effective medium theory models, the fitting procedures used, as well as the IR reflectance simulations using the time-domain solver of Computer Simulation Technology (CST) microwave studio.

### Data availability

The data that support the findings of this study are available from the corresponding authors (stephanie.gilbertcorder@stonybrook.edu and mengkun.liu@stonybrook.edu) upon request.

## Electronic supplementary material

Supplementary Information
