# Supplementary material for: Near-field spectroscopic investigation of dual-band heavy fermion metamaterials
Source: Nat Commun. 2017 Dec 22;8:2262. doi: 10.1038/s41467-017-02378-3 (PMC5741627; doi:10.1038/s41467-017-02378-3)
Supplement: Supplementary file 1 — Supplementary Information [file 41467_2017_2378_MOESM1_ESM.pdf]

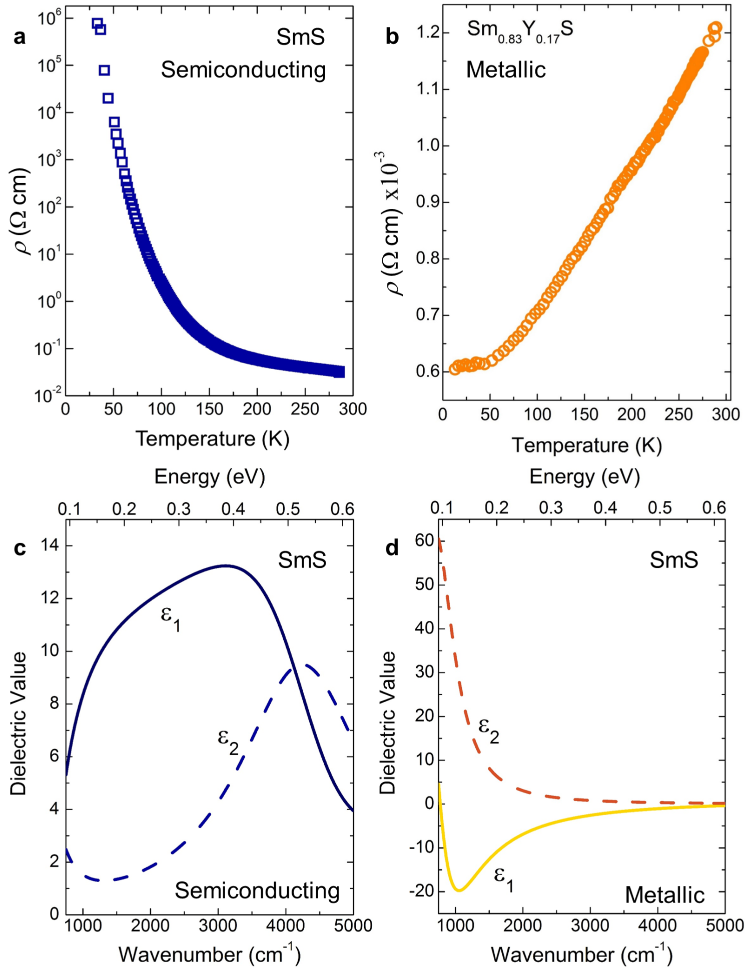

Supplementary Figure 1. **Material Properties**. (a) Temperature-dependent resistivity of the semiconducting SmS and (b) $Sm_{0.83}Y_{0.17}S$ before patterning. Real (ϵ_1) and imaginary (ϵ_2) dielectric values obtained from a Kramers-Kronig analysis of the unpatterned semiconducting SmS (c) and metallic (d) reflectivity shown in Figure 3b of the main text. The negative values of ϵ_1 in (d) for nearly the entire spectral window are consistent with the plasma resonance of the 4f electrons [1].

Supplementary Note 1: Material Properties

The single crystal materials were grown by the vertical Bridgman method in a high-frequency induction furnace in the same batch as those characterized by Imura, *et. al.* [2]. Further details on the growth process can be found in Matsubayashi, *et. al.* [3], along with the impact of controlled deviations in the stoichiometry and the resulting effects on the electrical properties. The resistivity of the as-grown semiconducting SmS and $Sm_{0.83}Y_{0.17}S$ samples used in these measurements can be seen in Supplementary Figure 1a and b as a function of temperature and indicate the high sample quality. At approximately 6.5 kbar

(P_{C1}), the undoped SmS undergoes a discontinuous (first order) phase transition from an optically black semiconductor to a golden intermediate valence (IV) state. The semiconducting color is the result of low reflectivity in the long wavelength portion of the visible region and strong optical transitions towards the blue part of the spectrum from $3p^6$ to the 5d t_{2g} states [4]. The optically-golden phase arises from a coupled plasmon mode with increasing reflectivity in the visible part of the spectrum [4, 5]. The golden IV phase can be achieved via polishing [4, 6] or applied pressure. We note that by simply pressing SmS with a fingernail, one can achieve ∼8 kbar pressure level thus induce the low pressure phase change in SmS. The dielectric values for the unpatterned semiconducting and golden IV phases can be see in Supplementary Figure 1c and d.

The Sm valence slowly increases with pressure [7]. This is a result of the 5d t_{2g} conduction band moving lower in energy with increasing applied pressure (crystal field splitting). As the conduction band moves lower, electrons in the 4f level spill into the conduction band until the 4f level is empty and the system is fully metallic with Sm^{3+} [8, 9]. Controversy exists over the band gap in golden IV SmS, with some claiming a pseudogap [3] and others a real gap. As stated in the text by Khomskii, golden IV SmS has a small gap: with pressure, the f levels cross the bottom of the initially empty conduction band and populate it with former f electrons. This creates an equal number of holes in the f states, which attract the conduction electrons, exhibit an exciton-like nature, and create a gap in the spectrum [9]. At high enough pressures, the exciton-like coupling between the 4f hole and the 5d electron may be destroyed as the electron bandwidth reaches a critical value and is no longer sufficiently localized; this has been stated as a possible origin of the IV transition [7]. Once the 4f state is empty of electrons (≥20 kbar, P_{C2}), the golden phase is considered metallic [3, 8].

When doped with substitutional yttrium (Y) ions to apply lattice pressure, the material properties of the resulting $Sm_{1-x}Y_xS$ vary significantly depending on the fraction of Y present, with the Y-containing materials are more amenable to pressure-induce deformations [10–13], as demonstrated in Supplementary Figure 2a and b. At a critical concentration around x=18%, the small band gap of $Sm_{1-x}Y_xS$ collapses as a result of the smaller ion [14]. The 4f electron levels are altered by the substitution: they merge with the conduction band at the critical concentration [15]. Thus, adding Y ions for Sm effectively removes f electron and adds d electrons [16]. The incorporation of Y decreases the lattice spacing, but maintains the rocksalt crystal structure of the SmS lattice, as seen in the XRD measurements

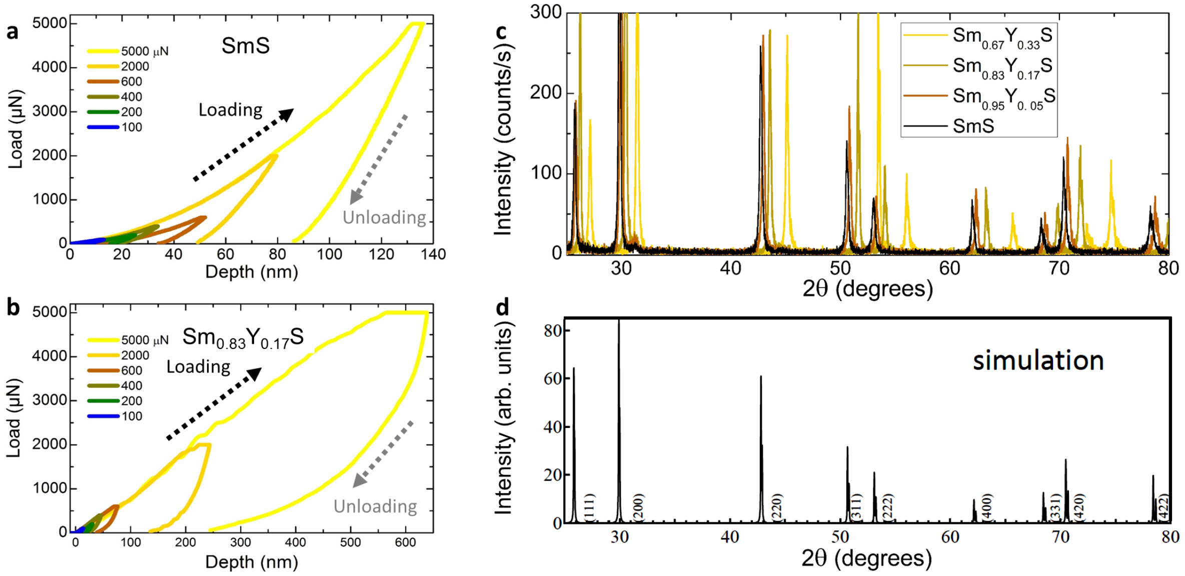

Supplementary Figure 2. **Material Characterization**. (a) Applied load vs. depth for nano-indention of the semiconducting black SmS and (b) $Sm_{0.83}Y_{0.17}S$, indicating the doped SmYS is significantly softer than the SmS. (c) XRD characterization of $Sm_{1-x}Y_x$ samples compared to simulated XRD peaks (d) for undoped SmS. The XRD shows that the lattice shrinks with increasing Y doping, but maintains the same crystal structure, consistent with previous reports [10–13].

of $Sm_{1-x}Y_xS$ in Supplementary Figure 2c. The simulated XRD results for semiconducting SmS can be seen in Supplementary Figure 2d. The XRD spectra indicate no other crystal structures are present in the materials, indicating high sample quality.

In both fully metallic SmS and $Sm_{1-x}Y_xS$, when the 4f electrons are no longer localized (either from pressure, dopant level, or a combination of the two), the IR resonance disappears and only the visible resonance remains. This is demonstrated in the main text.

Supplementary Note 2: Patterning

Many methods for nanoscale materials patterning via AFM exist—see for instance the review by Xie *et al.* [17]. To demonstrate the flexibility of the fabrication procedure, two methods were used in this work: (1) a brute-force approach using hard contact between the AFM tip and sample surface, where the scan is defined such that it makes the desired MM pattern without knowing the exact force/pressure applied (any AFM can be used) and (2) patterning based on the capabilities of AFM lithography software with controllable

tip-induced pressure. We note that three different AFMs were used for the lithography, demonstrating the general feasibility of the fabrication methods employed.

The fishnet and grating IR patterns investigated in Figures 2e, 3b and Supplementary Figure 4 were fabricated with a NeaSpec NeaSNOM AFM using the "brute-force" method, resulting in linewidths of 50–200 nm. The fishnet MM consists of a 20 x 20 lithographic grid with line spacing of 1 μm and a total area of approximately 40 μm x 40 μm. An optical image of the portion of the fishnet characterized in far-field FTIR measurements can be seen in Supplementary Figure 6. The grating MM is 10 parallel lines spaced 1 μm apart and connected at one end by a vertical line with a total area of approximately 12 μm x 12 μm. A near-field image of the grating characterized in near-field and far-field FTIR measurements can be seen in Supplementary Figure 4a.

The colour gradient MM shown in Figure 2a–d along with the red grating and golden grating MM patterns shown in Supplementary Figure 6 were made on a Park atomic force microscope (AFM) using the Park XEL software for lithography. A pre-defined pattern was created in the software and transferred to the SmS surface. The striped visible MM covers an area of 40 μm x 40 μm and the average spacing increases along the length from 163, 212, 266, 314, 363 to 412 nm with a depth of approximately 12 nm. The red grating MM covers a 30 μm x 30 μm area and the spacing between the lithographically patterned lines is 320 nm (average depth 12.5 nm) and 400 nm (average depth 11 nm). The golden grating MM is 30 μm x 30 μm in area, consisting of line spacing intervals of 230, 304, and 395 nm with average depths of 15, 6, and 8 nm, respectively.

The initials for Stony Brook University (SB in Figure 4d) were patterned using the lithography function of the NT-MDT AFM software. The letters cover a 3 μm x 3 μm area and were fabricated by tracing a pre-defined pattern in the lithography program. For thicker areas of the pattern, three parallel lines were used as indicated in the Figure 4d, 493 K image. The three parallel vertical lines above the letters were patterned (from left to right) using pressures of approximately 13.6 kbar (line traced three times), 13.6 kbar (line traced one time), and 8.9 kbar (line traced one time).

Supplementary Note 3: Applied Force and Residual Strain Estimates

Using the force-distance spectroscopy capability of the NT-MDT AFM, we are able to estimate the pressure range used to make the patterns as approximately 5–15 kbar, depending

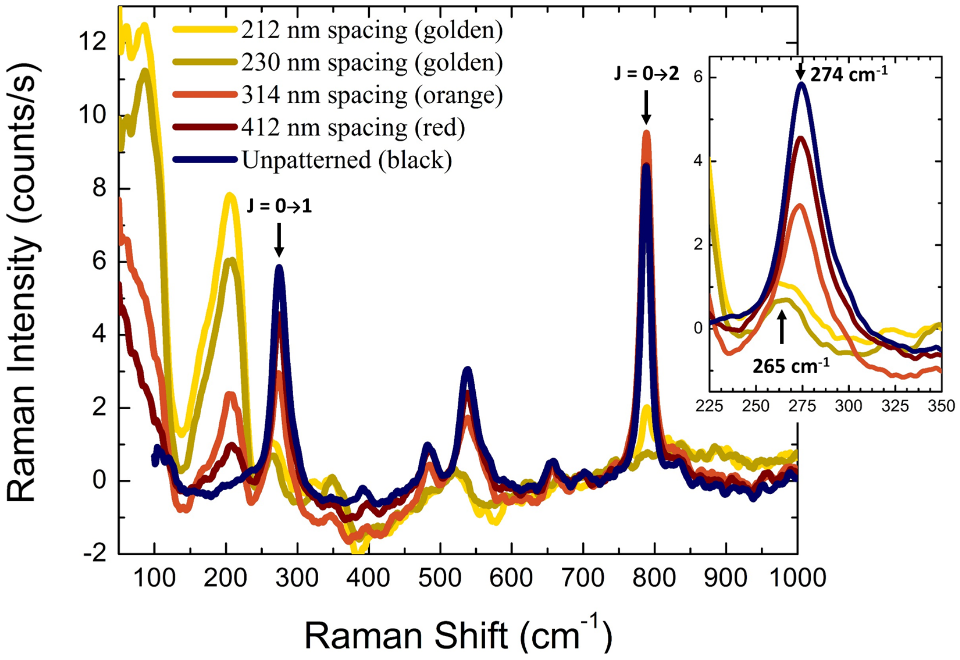

Supplementary Figure 3. **Raman Characterization**. Raman measurements for different spacings of the simple metamaterial grating on the undoped SmS. Peaks corresponding to the J transitions of the 4f states are observed, consistent with previous reports [18]. With increasing applied strain resulting from greater pattern density at the surface, the peaks redshift as shown in the inset. The estimated residual strain from the unpatterned black to the patterned golden spectra is estimated to be compressive on the order of 1.7%.

on the tip geometry. Raman spectroscopy was performed on the undoped SmS sample for different regions of the grating structures corresponding to an unpatterned (black) reference area, an optically red grating region with an average spacing of 412 nm, an optically orange region with a spacing of approximately 314 nm, and two golden regions with spacings of 230 nm and 212 nm as seen in Supplementary Figure 3. The excitation wavelength used was 532 nm with a 2 mW average power. The estimated spot size was 1.1 μm: given that the patterning linewidths are on the order of 50 nm, the spot size essentially measures both the patterned and unpatterned material within a given area. The Raman measurements on the patterns therefore inherently underestimate the amount of strain present since they sample a significant amount of the unpatterned material. Nevertheless, we observe a clear shift resulting from patterning. Supplementary Figure 3 highlights two specific transitions in semiconducting SmS; specifically, the J=0-1 and J=0-2 transitions around 274 cm^{-1} and 790 cm^{-1}, respectively, consistent with previous reports [18]. Comparing the J=0-1 transition for the black to golden phases, we observe a gradual shift from 274 cm^{-1} to 265 cm^{-1},

corresponding to increasing compressive strain with patterning up to 1.7%. The volume collapse values in three dimensions (hydrostatic) for the black to golden transition are generally reported to be between 6 and 15% (see [10, 19–21]). Considering these reported values are generally performed under hydrostatic pressure in three dimensions, whereas our strain measurements occur after the removal of applied pressure in one dimension and the Raman measurement samples an area containing both golden patterned lines as well as the black semiconductor between, the strain value determined from the Raman measurement is quite reasonable.

Supplementary Note 4: Near-field Characterization Methods

Infrared scattering-type scanning near-field optical microscopy (s-SNOM) is an AFM-based IR technique [22]. Combined with different light sources, s-SNOM naturally provides exquisite optical imaging and spectroscopy capabilities with a spatial resolution limited only by the apex radius of the AFM tip (∼20 nm in our case). In a typical setup, a broadband or CW laser beam is focused onto a metalized AFM tip, and the back-scattered signal (S_n) is demodulated at integral harmonics n=2, 3 of the tip-tapping frequency. The higher harmonic signals (S_2–S_3) reflect the strength of near-field coupling between the AFM tip and the sample surface, thus conveying information about the local dielectric function [23, 24].

Multiple near-field systems were used in this work and a schematic of the general setup can be seen in Figure 1c. A NeaSpec NeaSNOM system was used to conduct the near-field optical imaging shown in Figures 1c; 2c, e; 4c; and Supplementary Figure 2. The system uses an MCT detector and a tunable QCL as a source. The measurements reported in Figure 1c, 2e, and Supplementary Figure 4a were conducted at 188 meV or 6.6 μm. The imaging in Figure 2c at 1.7 eV or 730 nm and the imaging of Figure 4c at 1.68 eV or 738 nm were performed on a NeaSpec NeaSNOM system using a tunable Ti:sapphire laser as a source and a Si detector. The metallic signal was referenced to the semiconducting. The IR near-field imaging in Figure 2d, and Figure 4b, d, e, and f was performed on an NT-MDT NTEGRA-IR system with CO_2 laser as a sources (117 meV/10.6 μm or 112 meV/11 μm) and an MCT detector. The metallic signal was referenced to the semiconducting. The broadband near-field spectroscopy was conducted at the Advanced Light Source, Lawrence Berkeley National Laboratory with the synchrotron infrared nano-spectroscopy (SINS) system at

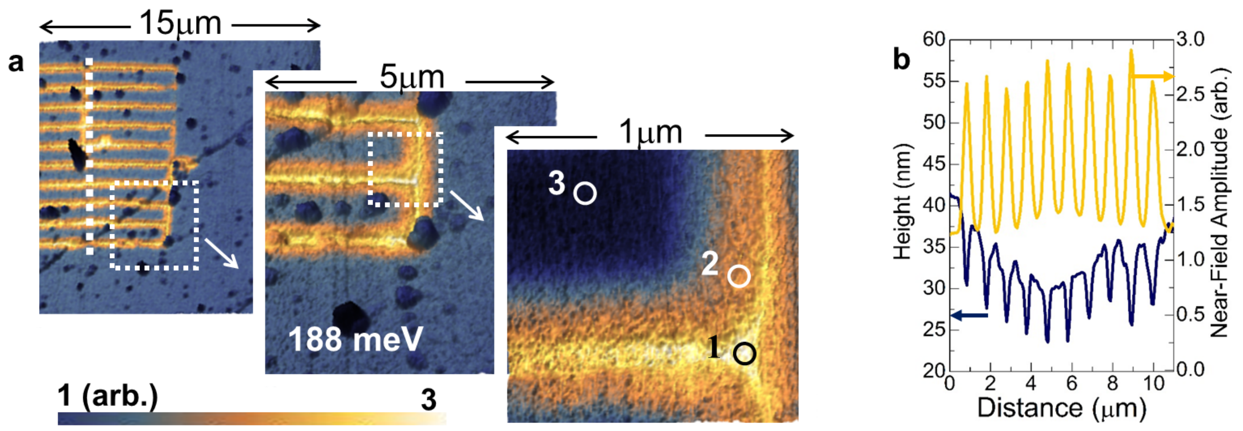

Supplementary Figure 4. **Near-field Characterization of MM**. (a) The near-field amplitude at 188 meV (1515 cm^{-1}) is shown in false colour for three levels of zoom (indicated by the white dotted boxes) showing the scale of the grating pattern. The near-field amplitude change of the patterned area (in gold) is normalized to the semiconducting response (in blue). Three locations are marked in the 1 μm image, indicating golden IV (1), transition (2), and unpatterned semiconducting (3) regions of the MM. (b) Topography (in blue) and corresponding near-field amplitude (in gold) measured along the vertical dashed line in the 15 μm image in (a), indicating the signal amplitude increase directly corresponds to the lithography.

beamline 5.4. A Ge:Cu detector was used for the measurements shown in Figure 2f and an MCT detector was used in Figure 2g and Supplementary Figure 5a. The synchrotron storage ring operated as a broad-band source and a rapid-scan FTIR was used to obtain frequency-resolved spectra. A gold thin film was used as a reference.

Supplementary Figure 4a shows the combined near-field amplitude (S_3) and topography image of a patterned SmS surface grating at 188 meV (1515 cm^{-1}). The boundary and the fine structure of the grating can be clearly seen in the 15 μm image, consisting of ten parallel lines spaced 1 μm apart. The white dashed boxes indicate the region probed with increasing full scale resolution of 5 and 1 μm. The images are shown in false-color where gold indicates the highly conductive regions and blue areas are semiconducting. It is clear from this image that surface dirt does not contribute to near-field signal. Supplementary Figure 4b shows a line-out of the sample topography (in blue) and near-field signal (in gold) corresponding to the vertical dashed line in the 15 μm image of Supplementary Figure 4a, revealing the conductive regions indeed correspond to the areas patterned lithographically. Variations in the near-field signal induced by topographic debris are negligible given the scale of the total change in the near-field amplitude.

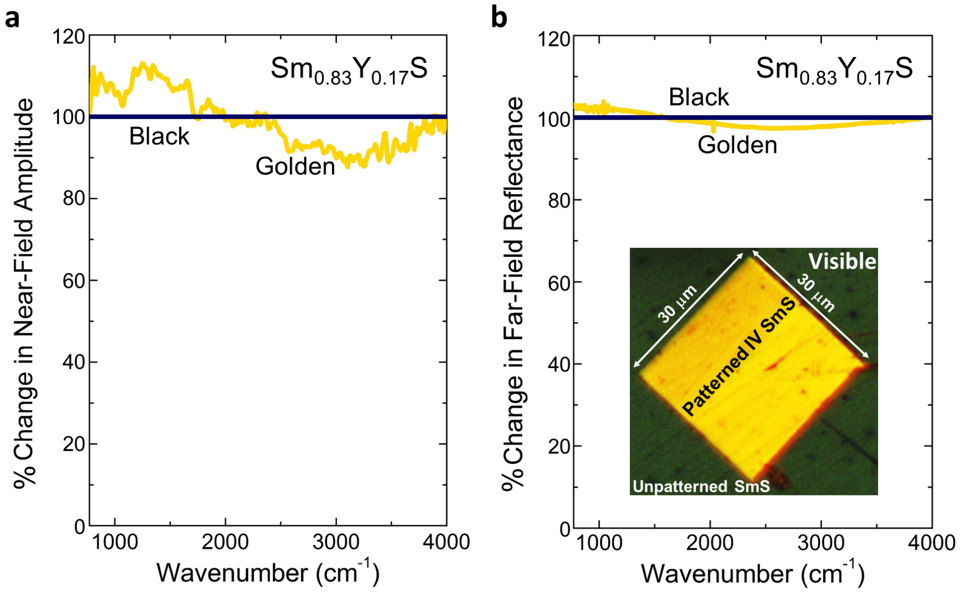

Supplementary Figure 5. **Characterization of $Sm_{0.83}Y_{0.17}S$ MM**. (a) The percent change in near-field amplitude is shown for a Y-doped sample where the patterned spectra has been normalized to the unpatterned response to show the relative change in signal. (b) The percent change in the far-field FTIR response, where the visibly golden patterned area has been normalized to the visibly black unpatterned area, consistent with the broadband IR near-field response which shows little change in this spectral region. Inset: Visible microscope image of the patterned region, showing the color change from the 5d electron band is still present with applied pressure.

The role of the AFM tip-induced strain on the phase change has a subtlety in that the sharpness of the tip effects the observed near-field response. For the gradient MM reported in Figure 2c at 412 nm, the lithographic lines are wider, possibly because the tip was dull by the end of patterning, producing a greater area over which the pressure was applied and resulting in the observed effect of less signal at 1.7 eV.

As an example of using chemical doping to control the strongly correlated 4f electron response, we use an $Sm_{1-x}Y_xS$ sample with x=17%, just below the critical value, where the black to golden phase transition can still be induced with pressure. A MM pattern is created on the sample surface using the same parameters as that of the golden grating MM in the undoped SmS. The resulting pattern is characterized using both near-field and far-field techniques, as illustrated in Supplementary Figure 5, where a shows the broadband near-field response collected on the golden patterned area and a black unpatterned area. The spectra are normalized to that of the black unpatterned area to highlight the lack of spectral change between the regions. Similarly, Supplementary Figure 5b shows the far-field FTIR spectra of 40 µm x 40 µm regions of the golden patterned and black unpatterned areas, normalized

to the unpatterned response. The inset in Supplementary Figure 5b is a visible microscope image of the same pattern, illustrating the black-golden visible color change. From these results and the near-field images of Figure 4b and c in the main text, it is clear that the response of the far IR 4f electrons to applied pressure has been effectively "turned off" with Y doping, while the response of the 5d band in the visible remains unchanged. It may be possible to further manipulate the response of the 4f electron resonance by using $Sm_{1-x}Y_xS$ with intermediate values of x between 0–17%, as optical spectroscopy of $Sm_{.95}Y_{.05}S$ showed evidence of the 4f plasma resonance weakened with respect to pure SmS, but still present [25].

The measurements shown in Figure 4d-f occur after controlled heat treatment in a furnace at the specified temperature. Once the sample has returned to room temperature, the near-field conductivity and the topography were recorded. The sample was heated to 625 K; however, upon cooling the exact location of the previously patterned region could not be located with certainty as the visible phase change had disappeared. This temperature is therefore omitted from the figure. Rogers *et. al.* have demonstrated quasi-thermal cycling of SmS thin films using XRD characterization [6]. They report that while the lattice expands with heat treatment and the optically black phase is recovered around 620 K, two 4f-5d peaks remain shifted from the original semiconducting lattice constant by about 0.02 eV, possibly the result of stress in the as-deposited films being removed with annealing. We therefore simply present temperature as a way to tune the strength (decrease the resonance peaks) of the patterned regions.

Supplementary Note 5: Optical Microscopy

The visible reflectivity data and images in Figure 3a and the Supplementary Figure 5b inset were collected using an optical microscope, internal source, and a fiber spectrometer. An aperture was used to limit the area of interrogation. The spectra are referenced to the spectrum of the internal source to eliminate artifacts.

Supplementary Note 6: Polarization-dependent FTIR

The broadband response of the fishnet and grating structures are further examined in Supplementary Figure 6 with a Bruker Hyperion 3000 microscope coupled to a Vertex V70

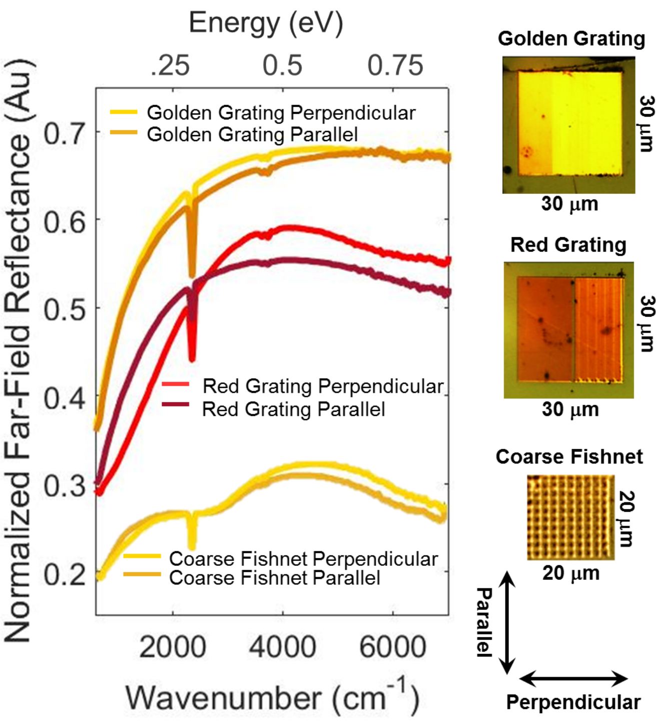

Supplementary Figure 6. **Polarization-dependent far-field FTIR of MM patterns**. The far-field reflectance is shown for the golden grating (optical microscope image, upper right), the red grating (optical microscope image, middle right), and a coarse fishnet structure (optical microscope image, lower right) referenced to a gold thin film. The grating patterns are approximately 30 μm x 30 μm in scale and the aperture used to collect the spectra was 20 μm x 20 μm in scale. The coarse fishnet is approximately 20 μm x 20 μm in scale, the same size as the aperture used to collect the spectra. All of the structures show resonance peaks that shift with polarization, indicating the presence of plasmonic MM effects.

FTIR at beamline 5.4 at the Advanced Light Source, Lawrence Berkeley National Laboratory. The polarization of the incident light relative to the MM patterns is controlled. The MM structures show a strong resonance near 5000 cm^{-1}, and a secondary peak is observed around 1800 cm^{-1} in the coarse fishnet structure shown in Supplementary Figure 6b. As the polarization of the incident light changes from perpendicular to parallel, the resonance peaks are observed to shift as a result of asymmetries in the patterns. The asymmetry in the coarse fishnet pattern is the result of a patterning artifact—the first set of parallel lines were fabricated with a significantly sharper tip, therefore they are likely to produce a stronger resonance than the subsequent set of parallel lines in the orthogonal direction.

The golden and red gratings are patterned as a series of vertical lines with different spacings (see the Patterning section for more detail). For the golden grating, the patterned regions are approaching fully metallic, which limits the shape effects as the 4f resonance

empties into the 5d conduction band in the fully metallic phase. The polarization dependent shift of the resonance is most notable in the red grating MM, however the spectra for both are markedly different from the coarse fishnet spectra, which shows two resonances rather than the single resonance of the gratings. Comparing the fishnet response to the red grating, the red grating shows a significantly stronger polarization dependence, as expected from the strong asymmetry. The polarization dependence, resonance features, and CST simulations indicate that although effective medium theory accurately determines the baseline reflectivity of the tip-engineered dual-band MM, plasmonic shape effects play an important role and can not be omitted when analyzing the IR response.

Supplementary Note 7: Simulated Far-Field IR Response with the Drude Model

The spectra in Figure 3a are fit using the Drude model of the dielectric function

$$\epsilon = (1 - \frac{\omega_p^2}{\omega^2}) + i(\frac{\omega_p^2}{\omega^3/\gamma}) \tag{1}$$

where ϵ is the semiconducting dielectric function, ω_p is the plasma frequency, ω is the frequency of the incident light, and γ is the damping term. The semiconducting damping value (1350 cm^{-1}) is used in the fitting of the red and golden spectra such that the only variable is the plasma frequency ω_p. The fully golden plasma frequency is reported to be 2.45 eV [4]. The values obtained from the fitting are 1.81 eV (14,600 cm^{-1}), 1.93 eV (15,550 cm^{-1}), and 2.16 eV (17,450 cm^{-1}) for the semiconductor, red grating, and golden grating spectra, respectively. The shifting plasma edge illustrates the influence of the effective carrier density change with pattern spacing on the observed optical response.

Supplementary Note 8: Simulated Far-Field IR Response with Effective Medium Theory

The effective medium simulations in Figure 3b were conducted using Lichtenecker's mixing rule, which describes the optical properties arising from the topology of composite systems [26–29]:

$$\epsilon_{eff}^k = (1 - f)\epsilon_{ins}^k + f\epsilon_{met}^k \tag{2}$$

Where ϵ_{eff} is the complex dielectric function of the composite medium, ϵ_{ins} is the complex dielectric function of the semiconducting phase, ϵ_{met} is the complex dielectric function of

the metallic phase, f is the fill fraction or amount of metallic phase present, and k is a depolarization factor which can range from -1 to 1. For spherical inclusions, $k = 1/3$; for cylindrical inclusions, $k = 1/2$; and for $k = \pm 1$, the composite is made of needle-like planes parallel to (+) or perpendicular (-) to the electric field. For the simulations shown here, the depolarization factor producing the best fit was -1/2, indicating inclusions which are cylindrical and anti-aligned with the incident light, consistent with the experimental parameters of the confocal microscopy far-field measurements. The effective medium theory results indicate that while the increase in baseline reflectivity observed in the patterned areas can be explained by the increase in metallic volume fraction, the peaks present are the result of plasmonic metamaterial effects, as explored below in the CST simulations.

Supplementary Note 9: Simulated Far-Field IR Response with CST Plasmonic Resonances

The full range of the IR reflectance simulated in the time-domain solver of Computer Simulation Technology (CST) microwave studio can be seen in Supplementary Figure 7a. The simulations use the far-field dielectric values plotted in Supplementary Figure 1c, and d. The fishnet is simulated using metallic lines which are 200 nm wide, 1 μm apart, and 300 nm deep to create a metallic pattern which is 12 μm x 12 μm on a semiconducting substrate—this is smaller than the actual pattern (measured area 20 μm x 20 μm) but saves computational time. The simulated fishnet reflectance response is therefore shifted up by 20% to more directly compare to the experimental data. The grating is simulated using metallic lines which are 200 nm wide, 1 μm apart, and 300 nm deep to create a metallic pattern which is 12 μm x 12 μm on a semiconducting substrate. The unpatterned semiconductor and metallic reference spectra are simulated as 20 μm x 20 μm featureless slabs. The width and depth of the simulations, which are larger than the physical lithography, may be an indication of the extent of the phase change in the material. In the IR region, the depth of the golden phase in the MM pattern—or how far the phase change extends into the substrate—is observed to strongly influence the resonance peak around 4250 cm^{-1}, as illustrated by Supplementary Figure 7b. The width and spacing are held constant at 0.5 μm and 1 μm, respectively. The peak increases with depth up to a maximum at 90 nm before decreasing to a baseline value. The drop in the resonance strength between 90 and 150 nm, and again between 300

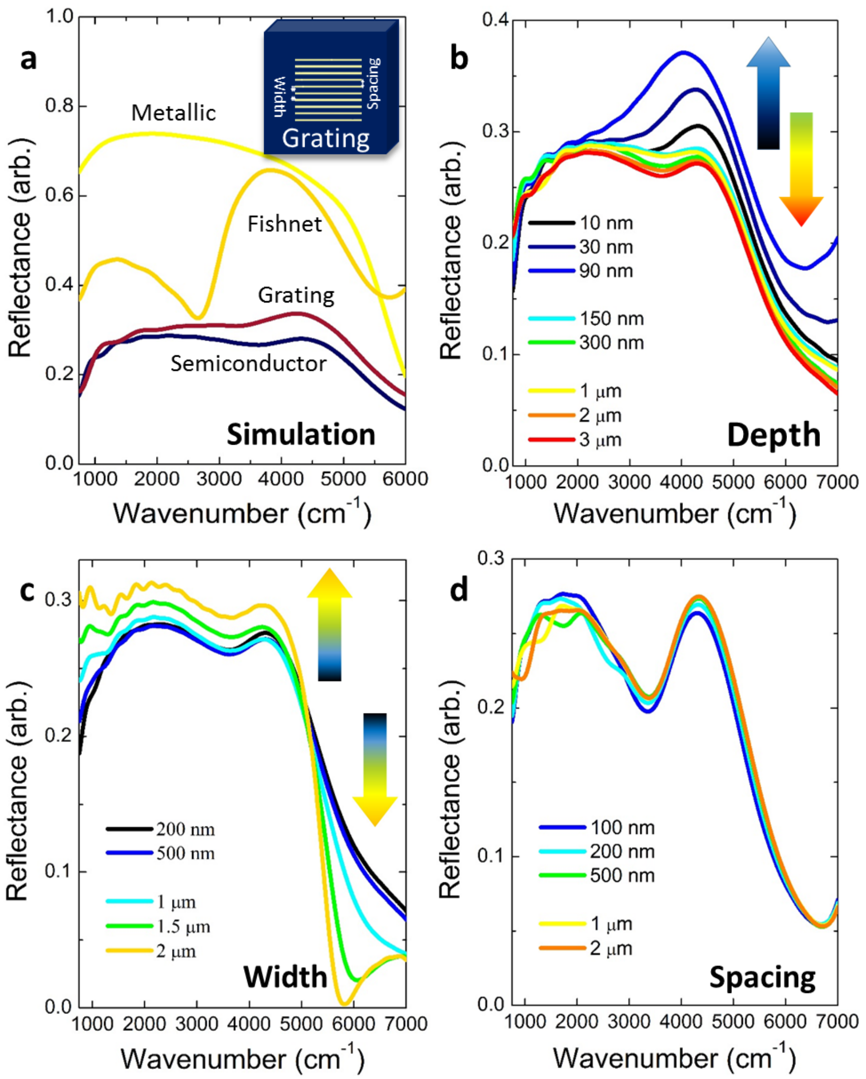

Supplementary Figure 7. **Simulations tuning of the IR plasmonic resonances** (a). The full range of the simulated reflectance response in the far-field. Varying the depth of the lithography (how far the golden phase change extends into the semiconductor host) is shown in (b) for the grating structure while holding the width and spacing constant. The resonance near 4500 cm^{-1} initially increases with depth before decreasing to a baseline value, suggesting an optimal region. (c) Altering the width of the golden inclusions while keeping the depth and spacing constant. The width is seen to increase the baseline reflectivity, as well as shift the edge of the IR plasma resonance to lower frequencies, possibly even achieving zero reflectivity near 5800 cm^{-1}. (d) Changing the line spacing while keeping the depth and metallic fill fraction constant produces no noticable effects on the response.

nm and 1 μm is due to a necessary change of the mesh settings. The simulation results have been separated into three groups for which the mesh settings were the same. The resonance near 4250 cm^{-1} increases with depth from 10 to 90 nm; decreases from 150 to 300 nm; and continues to decrease from 1 to 3 μm. The high energy tail around 6500 cm^{-1}

follows the same trend. The influence of the width of the metallic lines on the far-field result can be seen in Supplementary Figure 7c. The depth and spacing are held constant at 3 μm and 1 μm, respectively. The simulation results have been separated into two groups for which the mesh settings were the same. Altering the width effectively changes the fill fraction and thus increasing reflectivity, as well as shifting the plasma edge to lower frequencies. This simulation indicates it may be possible to achieve zero reflectivity near 5800 cm^{-1} for certain fabrication conditions. The effect of metallic line spacing on the far-field response is calculated in Supplementary Figure 7d while holding the depth and width constant at 3 μm and 0.5 μm, respectively. The simulation results have been separated into two groups for which the mesh settings were the same. The total metallic fill fraction is maintained during this simulation by changing the number of metallic lines. For a constant fill fraction, the spacing between the metallic lines doesn't appear to play a significant role in the plasmonic response. This is consistent with the near- and far-field results from the main text, which indicate spacing plays little role in the far IR resonance. From these results, we determined that the depth/applied pressure, width, and the metallic fill fraction determine the IR response of the SmS MM.

SUPPLEMENTARY REFERENCES

[1] Batlogg, B. & Wachter, P. Low energy anomalies in the optical spectrum of SmS with intermediate valence. *Valence instabilities and related narrow band phenomena* Parks, R.D., 537–540 *Plenum NY* (1977).

[2] Imura, K., *et al.* Origin of the black-golden transition in $Sm_{1-x}Y_xS$. *J. Phys.: Conf. Ser.* **592**, 012028 (2015) doi:10.1088/1742-6596/592/1/012028.

[3] Matsubayashi, K., *et al.* Effect of nominal composition on transport, optical, magnetic, and thermodynamic properties of SmS single crystals. *J. Phys. Soc. Jpn.* **76**, 064601, 6 (2007) doi:10.1143/JPSJ.76.064601.

[4] Batlogg, B., Kaldis, E., Schlegel, A. & Wachter, P. Electronic structure of Sm monochalcogenides. *Phys. Rev. B* **14**, 5503–5514 (1976) doi:10.1103/PhysRevB.14.5503.

[5] Travaglini, G. & Wachter, P. Low-energy electronic structure of intermediate-valence 'golden' SmS. *Phys. Rev. B* **30**, 5877–5883 (1984) doi:10.1103/PhysRevB.30.5877.

[6] Rogers, E., *et al*. The thermally induced metal-semiconducting phase transition of samarium monosulfide (SmS) thin films. *J. Phys. Condens. Matter* **22**, 015005, 7 (2010) doi: 10.1088/0953-8984/22/1/015005.

[7] Lapierre, F., Ribault, M., Flouquet, J. & Holtzberg, F. SmS and its alloys. *Journal of Magnetism and Magnetic Materials* **31**, 443 - 446 (1983) doi:10.1016/0304-8853(83)90314-1.

[8] Jarrige, I. , *et al*. Unified understanding of the valence transition in the rare-earth monochalcogenides under pressure. *Phys. Rev. B* **87**, 115107, (2015) doi:10.1103/PhysRevB.87.115107.

[9] Khomskii, D. Basic aspects of the quantum theory of solids. *Cambridge University Press* (2010).

[10] Varma, C. M. Mixed-valence compounds. *Rev. Mod. Phys.* **48**, 219–238, (1976) doi: 10.1103/RevModPhys.48.219.

[11] Coey, J. M. D., Ghatak, S. K., Avignon, M., & Holtzberg, F. Electronic configuration of samarium sulphide and related compounds: Mössbauer-effect measurements and a model. *Phys. Rev. B* **14**, 3744–3752 (1971) doi:10.1103/PhysRevLett.27.511.

[12] Alekseev, P. A., *et al*. Magnetic spectral response and lattice properties in mixed-valence $Sm_{1-x}Y_xS$ solid solutions studied with x-ray diffraction, x-ray absorption spectroscopy, and inelastic neutron scattering. *Phys. Rev. B* **74**, 035114, (2006) doi:10.1103/PhysRevB.74.035114.

[13] Parks, R. Valence Instabilities and Related Narrow-Band Phenomena. *Springer* (1977) doi: 10.1007/978-1-4615-8816-0.

[14] Pollack, R. A., Holtzberg, F., Freeouf, J. L. & Eastman, D. E. Temperature- and composition-dependent valence mixing of Sm in cation- and anion-substituted SmS observed by X-ray photoemission spectroscopy. *Phys. Rev. Lett.* **33**, 820–823 (1974) doi: 10.1103/PhysRevLett.33.820.

[15] Güntherodt, G., Jayaraman, A., Anastassakis, E., Bucher, E., & Bach, H. Effect of configuration crossover on the electronic Raman scattering by 4f multiplets. *Phys. Rev. Lett.* **46**, 855–858 (1981) doi:10.1103/PhysRevLett.46.855.

[16] Freeouf, J. L., Eastman, D. E., Grobman, W. D., Holtzberg, F., & Torrance, J. B. Spectroscopically observed valence mixing in SmS and related compounds. *Phys. Rev. Lett.* **33**, 161–164 (1974) doi:10.1103/PhysRevLett.33.161.

[17] Xie, X. N., Chung, H. J., Sow, C. H. & Wee, A. T. S. Nanoscale materials patterning and engineering by atomic force microscopy nanolithography. *Mat. Sci. Eng. R.* **54**, 1–48 (2006) doi:10.1016/j.mser.2006.10.001.

[18] Nathan, M. I., Holtzberg, F., Smith, J. E., Torrance, J. B. & Tsang, J. C. Electronic Raman Scattering and Infrared Absorption in the Samarium Monochalcogenides. *Phys. Rev. Lett.* **34**, 467–469 (1975) doi:10.1103/PhysRevLett.34.467.

[19] Maple, M. B. & Wohlleben, D. Nonmagnetic $4f$ Shell in the High-Pressure Phase of SmS. *Phys. Rev. Lett.* **27**, 511–515 (1971) doi:10.1103/PhysRevLett.27.511.

[20] Deen, P. P., *et al*. Structural and electronic transitions in the low-temperature, high-pressure phase of SmS. *Phys. Rev. B* **71**, 245118, (2005) doi:10.1103/PhysRevB.71.245118.

[21] Li, Z., Blaha, P. & Kioussis, N. Predicted topological phase transition in the SmS Kondo insulator under pressure. *Phys. Rev. B* **89**, 121117(R), (2014) doi:10.1103/PhysRevB.89.121117.

[22] Yang, H. U., Hebestreit, E., Josberger, E. E. & Raschke, M. B. A cryogenic scattering-type scanning near-field optical microscope. *Rev. Sci. Instrum.* **84**, 023701 (2013) doi: 10.1063/1.4789428.

[23] Jones, A. C., Berweger, S., Wei, J., Cobden, D. & Raschke, M. B. Nano-optical investigations of the metal-insulator phase behavior of individual VO_2 microcrystals. *Nano Lett.* **10**, 1574–1581 (2010) doi:10.1021/nl903765h.

[24] Huber, A. J., Ziegler, A., Köck, T. & Hillenbrand, R. Infrared nanoscopy of strained semiconductors. *Nat. Nanotech.* **4**, 153–157 (2009) doi:10.1038/NNANO.2008.399.

[25] Güntherodt, G., & Holtzberg, F. Optical studies of semiconductor to metal transition in Sm1-xYxS. *AIP Conf. Proc.* **24**, 36–37 (1975) doi:10.1063/1.30135.

[26] Theiß, W. Optical properties of porous silicon. *Surf. Sci. Rep.* **29**, 91, 101 (1997) doi: 10.1016/S0167-5729(96)00012-X.

[27] Looyenga, H. Dielectric constants of heterogeneous mixtures. *Physica* **31**, 401, 5 (1965) doi:10.1016/0031-8914(65)90045-5.

[28] Zakri, T., Laurent, J.-P. & Vauclin, M. Theoretical evidence for 'Lichtenecker's mixture formulae' based on the effective medium theory. *J. Phys. D: Appl. Phys.* **31**, 1589–1594 (1998) doi:10.1088/0022-3727/31/13/013.

[29] Goncharenko, A. V., Lozovski, V. Z. & Venger, E. F. Lichtenecker's equation: applicability and limitations. *Opt. Commun.* **174**, 19–32 (2000) doi:10.1016/S0030-4018(99)00695-1.
